# Binary choice games and arithmetical comprehension

**DOI:** 10.1007/s00153-026-01016-4

**Published:** 2026-05-09

**Authors:** J. P. Aguilera, T. Kouptchinsky

**Affiliations:** Institute of Discrete Mathematics and Geometry, Vienna University of Technology, Wiedner Hauptstrasse 8-10, 1040 Vienna, Austria

**Keywords:** Reverse mathematics, Determinacy, Weak König’s lemma, Primary 03B30, Secondary 03F35

## Abstract

We prove that Arithmetical Comprehension is equivalent to the determinacy of all clopen integer games in which each player has at most two moves per turn.

## Introduction

Do infinite games in the Cantor Space ($2 ^{\omega }$) have winning strategies? In $\textsf{ZFC}$, the answer does not differ from the analogous situation in the Baire space ($\omega ^{\omega }$), and Martin has proven that such games with Borel payoff sets are determined. Reverse mathematics ([4]) utilizes weak axiom systems ruling second order arithmetic definitions to highlight differences in provability strength between theorems formalizable in this setting. For instance, over $\textsf{RCA}_0$, a well known theorem of Steel [5] shows determinacy for clopen games in $\omega ^{\omega }$, $\Delta ^0_1$-$\textsf{Det}$, requires the use of transfinite recursion ($\textsf{ATR}_0$). Nemoto, MedSalem, and Tanaka [2] have shown that the same statement for clopen games over $2^{\omega }$, termed $\Delta ^0_1$-$\textsf{Det}^*$, only requires the statement of weak König’s lemma to be satisfied ($\textsf{WKL}_0$), that is, a substantially weaker subsystem.

The study of these games in second order arithmetic highlights how restricting games to a compact space reduces the necessary set existence axioms on which relies the existence of winning strategies. Simpson [4] has shown that the determinacy of all games of length *k* with integer moves is equivalent to $\textsf{ACA}_0$ (for a fixed $k \ge 3$), thus placing determinacy of finite games with infinitely many moves in between $\Delta ^0_1$-$\textsf{Det}^*$ and $\Delta ^0_1$-$\textsf{Det}$.

As $\textsf{WKL}_0$ states that every infinite tree of $2^{\omega }$ has an infinite branch, König’s lemma, the analogous statement on infinite, but finitely branching trees of $\omega ^{\omega }$ is equivalent to $\textsf{ACA}_0$, provably over $\textsf{RCA}_0$. The same applies if we restrict König’s lemma to infinite trees for which all nodes have at most two successors. In the scope of this article, we call these trees binary choice game tree. Although the set of paths in a binary choice game tree is compact, it allows more information coded within, as the comparison between weak and regular König’s lemma suggests.

Therefore, the question arises whether allowing play in such arbitrarily coded compact spaces influences the set existence axioms involved in proving the existence of winning strategies for such games. The purpose of this article is to answer this question.

In the games we consider, we suppose that players alternate turns playing numbers $x_i \in \mathbb {N}$. After infinitely many moves have been played, the winner is determined based on whether $x = (x_0, x_1, x_2, \hdots )$ belongs to some clopen subset of $\mathbb {N}^\mathbb {N}$. The additional condition is that in each turn, each player has at most two legal moves they can make, with the provision that an illegal move results in losing the game. The games are defined more carefully in §2 using the mechanism of *game trees*: the game *G*(*T*, *A*) is the game in which player I’s winning set is *A*, but both players are required to play within the tree *T*. Our answer is as follows.

### Theorem 1.1

Fix $k \ge 1$. The following are equivalent over $\textsf{RCA}_0$:

1. Determinacy for binary choice wellfounded game trees.
2. $\Delta ^0_1$-$\textsf{Det}$ for binary choice game trees.
3. $(\Sigma ^0_1)_k$-$\textsf{Det}$ for binary choice game trees.
4. $$\textsf{ACA}_0$$.

Here $(\Sigma ^0_1)_k$ denotes the *k*th level of the difference hierarchy over $\Sigma ^0_1$, i.e., the class of sets obtained from *k* nested differences of open sets.

As we exposed, theorem 1.1 is to be contrasted with previous results concerning similar games. Here is a non-exhaustive summary of the proof-theoretic strength of similar games.

**Table:** 

Provability strength of statements of determinacy			
Subsystem	Cantor Space	Binary branching game trees	Baire space
$\textsf{WKL}_0$	$\Delta ^0_1$-$\textsf{Det}^*$, $\Sigma ^0_1$-$\textsf{Det}^*$ [2]		
$\textsf{ACA}_0$	$(\Sigma ^0_1)_2$-$\textsf{Det}^*$ [2]	Wellfounded	Games of length
		games, $\Delta ^0_1$ games,	*k*, for $k \ge 3$ [4]
		$(\Sigma ^0_1)_k$ games for	
		$$k \ge 1$$	
$\textsf{ATR}_0$	$\Delta ^0_2$-$\textsf{Det}^*$, $\Sigma ^0_2$-$\textsf{Det}^*$ [2]		$\Delta ^0_1$-$\textsf{Det}$, $\Sigma ^0_1$-$\textsf{Det}$ [5]
$\Pi ^1_1$-$\textsf{CA}_0$	$\textrm{Sep}(\Sigma ^0_1, \Sigma ^0_2)$-$\textsf{Det}^*$ [3]		$(\Sigma ^0_1)_{2}$-$\textsf{Det}$ [6]

Our proof will make clear that $\textsf{ACA}_0$ allows translating from arbitrary binary choice game tree to a game in the Cantor space, with equivalent complexity, beyond the difference hierarchy over open payoff sets. Similarly, determinacy in Cantor and Baire space coincide for classes closed under recursive substitutions, unions with $\Sigma ^0_2$ sets, and intersections with $\Pi ^0_2$ sets.

In what follows we assume some basic knowledge of subsystems of second-order arithmetic, e.g., as presented in Simpson [4]. Note that we allow the formulae in our classes to possibly contain second-order parameters.

## Binary choice games in second-order arithmetic

### Definition 2.1

*(Notations)* We denote by $\mathbb {N}\text{}^{\textrm{even}}$ (respectively $\mathbb {N}\text{}^{\textrm{odd}}$) the subset of $\mathbb {N}\text{}^{<\mathbb {N}\text{}}$ consisting only of finite sequences of even (respectively odd) length. Given $x \in \mathbb {N}\text{}^{<\mathbb {N}\text{}}$ and $n < \textrm{length}(x)$ we write *x*[*n*] for the finite sequence $\langle x(0), \cdots , x(n-1) \rangle$. Given *S* a subset of $\mathbb {N}\text{}^{<\mathbb {N}\text{}}$ and $x \in S$, we write $S_{x}$ for the set of $y \in \mathbb {N}\text{}^{<\mathbb {N}\text{}}$ such that $x^{\smallfrown }y \in S$, finally, we say that *S* is bounded if it contains finitely many elements.

### Definition 2.2

*(Binary choice tree)* The set *T* of (codes for) finite sequences of natural numbers $(\tau \in \mathbb {N}\text{}^{< \mathbb {N}\text{}})$ is a binary choice tree if it satisfies:

1. $$\forall \tau \ \tau \in T \rightarrow (\forall n < |\tau |)\ \tau [n] \in T$$ and;
2. $$\forall \tau \ \tau \in T \rightarrow \exists ^{\le 2} n \ \tau ^\smallfrown n \in T$$.

Note that we therefore allow binary choice trees to have no branches. For instance, any subtree of $2^{\mathbb {N}}$ is a binary choice tree, or even

$$\begin{aligned} \{ \langle \rangle , \langle 0 \rangle , \langle 3435 \rangle , \langle 0, 1\ 047\ 527\ 295\ 416\ 280 \rangle \}. \end{aligned}$$

### Definition 2.3

Let *T* be a binary choice game tree.

1. We define a regular strategy $\sigma$ (respectively, $\tau$) for player $\textrm{I}$ (respectively, $\textrm{II}$) – written $\sigma \in \textrm{S}_{\textrm{I}}$ (respectively $\tau \in \textrm{S}_{\textrm{II}}$) – as any function $\sigma : \mathbb {N}\text{}^{\textrm{even}} \rightarrow \mathbb {N}\text{}$ (respectively $\tau : \mathbb {N}\text{}^{\textrm{odd}} \rightarrow \mathbb {N}\text{}$). We define $\sigma \otimes \tau$ as the unique sequence of natural numbers $\langle n_{i} \rangle _{i \in \mathbb {N}}$ produced by the alternation of these two strategies across the even and odd indices. That is,
  $$\begin{aligned} n_{0} = \sigma (\emptyset ) \wedge n_{i+1} = \left\{ \begin{array}{ll} \sigma (\langle n_0, \dots , n_i \rangle ) & \text {if { i} is odd}; \\ \tau (\langle n_0, \dots , n_i \rangle ) & \text {if { i} is even}. \end{array} \right. \end{aligned}$$
2. We define a restricted strategy $\sigma$ for player $\textrm{I}$ in the binary choice game tree *T* – written $\sigma \in \textrm{S}_{\textrm{I}}(T)$ – as a subtree of *T* that satisfies the properties:
  $$\begin{aligned}&\forall t \in \mathbb {N}\text{}^{\textrm{even}} \ (t \in \sigma ) \rightarrow (\exists ! n \ (t^{\smallfrown }n \in \sigma )) \quad \text {and} \\&\forall t \in \mathbb {N}\text{}^{\textrm{odd}} \ (t \in \sigma ) \rightarrow [\forall n \ (t^{\smallfrown }n \in T) \rightarrow (t^{\smallfrown }n \in \sigma )]. \end{aligned}$$
  A restricted strategy $\tau$ for player $\textrm{II}$ – written $\tau \in \textrm{S}_{\textrm{II}}(T)$ – is defined similarly, swapping the role of odd and even. We define $\sigma \otimes \tau$ as the intersection $\sigma \cap \tau$.

### Remark 2.4

For any binary choice game tree *T* and any node $t \in T$, $\textsf{RCA}_0$ proves that $Succ(t) = \{ s: \exists n \ s = t^{\smallfrown }n \wedge s \in T \}$ exists by bounded $\Sigma ^0_1$ comprehension (see Simpson [4]*theorem II.3.9).

The subtlety of games on binary choice trees relies on the fact that the exact structure of the game tree is inaccessible in $\textsf{RCA}_0$. Even if *T* is recursive, the set of nodes in *t* that have exactly one successor might not be. In contrast, for binary games with moves in $\{0,1\}$, and unrestricted games on natural numbers, the game tree can be assumed to be the full tree of their respective space. For these games, the winning condition is the only interesting structure to take into account.

### Definition 2.5

*(Finite difference hierarchy)* The class $(\Sigma ^0_1)_k$ of formulas is defined by recursion for $k \in \omega$.

- For $$k = 1$$, $$(\Sigma ^0_1)_1 = \Sigma ^0_1$$;
- For $k > 1$, $\phi \in (\Sigma ^0_1)_k$ iff there are $\psi _1, \psi _2$ such that $\phi \leftrightarrow \psi _1 \wedge \psi _2$, $\lnot \psi _1 \in (\Sigma ^0_1)_{k-1}$, and $\psi _2 \in \Sigma ^0_1$-

It can be shown$\cdot$that for any formula $\phi$ in the class of Boolean combinations of $\Sigma ^0_1$ formulas, there is $k \in \omega$ such that $\phi \in (\Sigma ^0_1)_k$.

Coming back to our binary choice game trees: we want to penalize the first player playing outside a tree *T*. To this aim, let us define $\mu _I(x)$, ranging over $\mathbb {N}\text{}^{\mathbb {N}\text{}}$ by the formula

$$\begin{aligned}&\exists n \ x[2n+1] \not \in T \wedge x[2n] \in T, \end{aligned}$$

that is, $\mu _I$ asserts that player $\textrm{I}$ played outside *T* first. The formula $\mu _{II}(x)$ is defined similarly, by

$$\begin{aligned}&\exists n \ x[2n+2] \not \in T \wedge x[2n+1] \in T. \end{aligned}$$

Taking $\phi (x) \in \Gamma$, we can then define:

### Definition 2.6

$\Gamma$-Determinacy for binary choice game trees asserts that whenever *T* is a binary choice game tree and $\phi \in \Gamma$, then the game $G(T,\phi )$ is determined, i.e.,

$$\begin{aligned} \exists \sigma \in \textrm{S}_{\textrm{I}}\forall \tau \in \textrm{S}_{\textrm{II}}\psi ( \sigma \otimes \tau ) \vee \exists \tau \in \textrm{S}_{\textrm{II}}\forall \sigma \in \textrm{S}_{\textrm{I}}\lnot \psi ( \sigma \otimes \tau ), \end{aligned}$$

where $$\psi (x) \equiv \lnot \mu _{\textrm{I}}(x) \wedge (\phi (x) \vee \mu _{\textrm{II}}(x))$$.

### Remark 2.7

If $\phi \in \Gamma$, $G(T, \psi )$ has a $\Pi ^0_1\wedge (\Gamma \vee \Sigma ^0_1)$ payoff set, but this payoff set is of a very particular kind, as the $\Pi ^0_1$ set and the $\Sigma ^0_1$ are both derived from the same tree *T*. For the classes $\Gamma$ we consider below (namely, $\Gamma = \Delta ^0_1$ or $\Gamma = (\Sigma ^0_1)_n$), this payoff set will generally be of complexity greater than $\Gamma$. By [1, Corollary 21], these are essentially the only classes for which the complexity differs.

Let us state an equivalent definition, for which the geometry of the tree does not impact the complexity of the winning condition.

### Definition 2.8

Let $\phi (x)$ be a formula of some class $\Gamma$ with a distinguished free set variable and *T* be a binary choice game tree. We say that the game $G(T,\phi )$ is determined in restricted strategies if

$$\begin{aligned} \exists \sigma \in \textrm{S}_{\textrm{I}}(T) \forall \tau \in \textrm{S}_{\textrm{II}}(\sigma ) \phi (\sigma \otimes \tau ) \vee \exists \tau \in \textrm{S}_{\textrm{II}}(T) \forall \sigma \in \textrm{S}_{\textrm{I}}(\tau ) \lnot \phi ( \sigma \otimes \tau ). \end{aligned}$$

We observe that in some cases, $\textrm{I}$ can have a winning strategy that prohibits $\textrm{II}$ from having any strategy, that is, that forces $\textrm{II}$ to play out of the tree, vacuously satisfying the winning condition this way.

### Remark 2.9

Definitions 2.6 and 2.8 are equivalent in $\textsf{RCA}_0$. That is for any formula $\phi$, and binary choice tree *T*, the game $G(T, \phi )$ is determined in the sense of 2.6 iff it is determined in the sense of 2.8, provably in $\textsf{RCA}_0$.

We introduce now a notion of determinacy for wellfounded binary branching trees.

### Definition 2.10

Let *T* be a wellfounded tree and *u* be a finite sequence of natural numbers. We define

$$\begin{aligned}&W_{\textrm{I}}(u) = \exists (n< |u|) \ u[n] \not \in T \wedge (\forall k< n) u[k] \in T \wedge n \text { is even}; \\  &W_{\textrm{II}}(u) = \exists (n< |u|) \ u[n] \not \in T \wedge (\forall k < n) u[k] \in T \wedge n \text { is odd}. \end{aligned}$$

We then say that the game on *T* is determined iff

$$\begin{aligned} \exists \sigma \in \textrm{S}_{\textrm{I}}\forall \tau \in \textrm{S}_{\textrm{II}}\ W_{\textrm{I}}(\sigma \otimes \tau ) \vee \exists \sigma \in \textrm{S}_{\textrm{II}}\forall \tau \in \textrm{S}_{\textrm{I}}\ W_{\textrm{II}}(\sigma \otimes \tau ). \end{aligned}$$

Notice that $W_{\textrm{I}}(\sigma \otimes \tau )$ can be expressed in a $\Delta ^0_1$ fashion since *T* is assumed to be wellfounded. Indeed, it can be expressed equivalently in the following ways

$$\begin{aligned} \exists n \ W_{\textrm{I}}((\sigma \otimes \tau )[n]), \text { and } \end{aligned}$$

$$\begin{aligned} \forall n \ (\sigma \otimes \tau )[n] \not \in T \rightarrow W_{\textrm{I}}((\sigma \otimes \tau )[n]). \end{aligned}$$

Therefore, determinacy for wellfounded binary trees is a suitable a priori weaker notion than $(\Sigma ^0_1)_k$-$\textsf{Det}$ for binary choice game trees, for $k \ge 2$.

## Proof of Theorem 1.1

### Theorem 3.1

Fix $k \ge 2$. The following are equivalent over $\textsf{RCA}_0$:

1. Determinacy for *wellfounded* binary choice game trees.
2. $\Delta ^0_1$-$\textsf{Det}$ for binary choice game trees.
3. $(\Sigma ^0_1)_k$-$\textsf{Det}$ for binary choice game trees.
4. $$\textsf{ACA}_0$$.

### Proof

First, we fix $k \ge 1$, assume $\textsf{ACA}_0$, and prove that $(\Sigma ^0_1)_k$-$\textsf{Det}$ for binary choice game trees holds. Given a binary choice game tree *T*, we define an embedding $\rho : T \rightarrow 2^\mathbb {N}$ by: $\rho (s^\smallfrown n) = \rho (s)^\smallfrown 0$ if $s^\smallfrown n$ is the leftmost successor of *s* in *T* and $\rho (s^\smallfrown n) = \rho (s)^\smallfrown 1$ if $s^\smallfrown n$ is a successor of *s* in *T* but not the leftmost successor. Observe that $\rho$ is recursive in *T*. By $\textsf{ACA}_0$, the tree $T' = \text {range}(\rho )$ exists as a set and is a subtree of $2^\mathbb {N}$. By Remark 2.7, every $(\Sigma ^0_1)_k$ game on the tree $T'$ belongs to the class $(\Sigma ^0_1)_{k+2}$ (as a subset of the full binary tree) and thus is determined, according to the theorem of Nemoto, MedSalem, and Tanaka [2]. Indeed, one can see their proof adapts to $\textsf{ACA}_0\vdash (\Sigma ^0_1)_{k}$–$\textsf{Det}^*$ for all $k \ge 2$. Consider $k>2$ and $\phi$ of the form $\exists n R(f[n]) \wedge \psi (f)$, where *R* is $\Pi ^0_0$ and $\psi$ is $(\Sigma ^0_1)_{k-1}$. We define *W* as:

$$\begin{aligned} W = \{ u \in 2^{< \mathbb {N}} : \text {there exists } v \subseteq u \text { such that } R(v) \text { holds and } \textrm{I}\text { wins } \psi (f) \text { at } u\}. \end{aligned}$$

It is easy to see, following their argument, that this set is arithmetical so that *W* exists in $\textsf{ACA}_0$ and the game winning condition $\exists n f([n]) \in W$ is $\Sigma ^0_1$. Thus, it forms an auxiliary game that is determined and yields the determinacy of the original game as argued by their proof. Using $\rho$ and $\textsf{ACA}_0$, a winning strategy for this game can be pulled back into a winning strategy for the game on *T*.

Conversely, since $(\Sigma ^0_1)_2$ for binary choice trees implies $(\Sigma ^0_1)_2$ for subtrees of $2^{\mathbb {N}}$, it follows from [2] that 3. implies 4.

Trivially, 3. implies 2., which implies 1. To complete the proof of the theorem, we show that $\textsf{ACA}_0$ follows from determinacy for wellfounded binary choice game trees. We appeal to the following well known result (see Simpson [4]*theorem III.7.2): $\textsf{ACA}_0$ is equivalent to weak König’s lemma for binary choice trees.

Thus, we suppose for the sake of a contradiction that there is an infinite binary choice tree *T* that does not have any branch. We will suppose without loss of generality that *T* does not contain any zeroes in its constitutive sequences. Otherwise, we just replace *T* by the tree $T'$ defined by

$$\begin{aligned} \langle n_0, n_1, \cdots n_i \rangle \in T' \leftrightarrow \bigwedge _{j = 0}^i (1 \le n_i) \wedge \langle n_0-1, n_1-1, \cdots n_i-1 \rangle \in T, \end{aligned}$$

which is also binary branching, also violates weak König’s lemma, and exists by $\Delta ^0_1$-Comprehension.

Let us first explain the game we consider, then we will show it has a wellfounded binary choice game tree, and finally we show how to obtain the conclusion from it. *The binary choice game tree will be different from* *T*.

The game is divided in four phases. During phase 1, player $\textrm{I}$ can construct any sequence $t \in T$. Then, during phase 2, player $\textrm{II}$ answers with $u_0\in \mathbb {N}$ with $t^{\smallfrown } u_0 \in T$. In phase 3, player $\textrm{I}$ produces a sequence *v*, intending to extend *t*, with $v(0) \ne u_0$. Finally, during phase 4, player $\textrm{II}$ produces a sequence $u'$, intending to extend $t^{\smallfrown }u_0$. Player II’s goal is for her sequence to be longer than $\textrm{I}$’s.
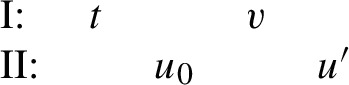


We now describe how to encode this game with a binary choice game tree. We explain how player I plays the sequence *t*; the sequences *v* and $u'$ are played similarly. Suppose a sequence $s \in T$ has been constructed so far by player $\textrm{I}$, and they would like to continue extending *s*. Then

A. First, player $\textrm{I}$ plays *n*, the leftmost successor of *s* in *T*.
B. As a second move, $\textrm{I}$ plays either 0 to confirm that *n* is the next element to be added to the sequence (recall that by choice of *T*, we have $s^{\smallfrown }0 \not \in T$), or else $\textrm{I}$ plays the rightmost successor *m* of *s* in *T*. In this case, *m* is added as the next element of the sequence.

The new sequence is now $s^{\smallfrown }n$ or $s^\smallfrown m$. After this, $\textrm{I}$ either indicates that they are not done constructing their sequence by playing 0 and repeat the above process, or plays 1 to indicate that the next phase of the game is to start. Throughout this process, player $\textrm{II}$ must play 0 each turn, as her moves are irrelevant.

Observe that throughout the construction of the sequence *t*, each player has at most two legal moves each turn. Moreover, whether a play by $\textrm{I}$ is legal requires checking finitely many instances of membership in *T*, so the binary choice game tree defined thus far is recursive.

Here is an illustration.
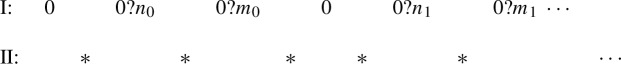

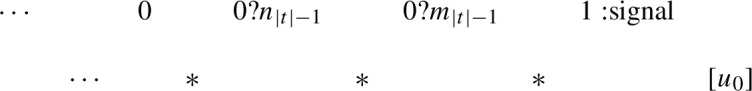


For example, here *t* is given by $\langle p_0, p_1, \cdots p_{|t|-1} \rangle$. The symbol $*$ indicates that player $\textrm{II}$’s moves are not relevant, although we formally require that she play zeroes. The play $u_0$ and the sequences *v* and $u'$ are encoded similarly using *T*, *except* that when playing $u_0$, we allow $n = 0$ in (A), in which case the next play by $\textrm{II}$ must also be 0 (confirming the choice $n = 0$). This is allowed if player $\textrm{II}$ believes *T* is not extendible on *t*, in which case they effectively challenge $\textrm{I}$ to provide a counterexample. We observe that player $\textrm{I}$’s legal moves require that $t^\frown v \in T$, and player $\textrm{II}$’s legal moves (given by the game tree) require that $t^\smallfrown u \in T$, where $u = \langle u_0 \rangle ^{\smallfrown }u'$, except if $u_0 = 0$.

The winning condition for $\textrm{II}$ is the set consisting of all plays satisfying:

3.1 $$\begin{aligned} v(0) = u_0 \vee v = \emptyset \vee t^{\smallfrown }v \not \in T \vee \big ( |v| \le |u| \wedge t^\smallfrown u \in T\big ). \end{aligned}$$

Since *T* has no branches by hypothesis, there are no infinite plays of the game, so it is indeed a wellfounded binary choice game tree.

According to the definition of games on binary choice game trees, we have the following: if one player plays a move outside of the game tree, then the first one to do so loses. Otherwise, if both players play within the game tree, player $\textrm{II}$ wins if and only if (3.1) holds. By construction of the game tree, we have the following:

i. If $t^\smallfrown v \not \in T$, then player $\textrm{II}$ wins;
ii. else, if $t^\smallfrown v \in T$ but $v = \emptyset$ or $v(0) = u_0$, then player $\textrm{II}$ wins;
iii. else, if $t^\smallfrown u \not \in T$, then player $\textrm{I}$ wins;
iv. else player $\textrm{II}$ wins if and only if $|v| \le |u|$.

Let us show that $\textrm{I}$ cannot have a winning strategy. Suppose otherwise and let $\sigma$ be a winning strategy. Let us consider *t*, the sequence $\sigma$ constructs in the first phase of the game. We must have that $t \in T$, as otherwise $\textrm{II}$ would win because of (i). We also must have that *t* has a successor *n* in *T*, otherwise $\textrm{II}$ would play $n = 0$ and win because of (ii). Since $\sigma$ is winning, *t* must be splitting, i.e., have two immediate successors *n* and *m*, for if it only had one, it could be chosen as $u_0$ and player $\textrm{II}$ would win by (ii).

Let $T_n = \{ s: t^{\smallfrown }n^{\smallfrown }s \in T \}$ and $T_m$ be defined similarly. There are two cases:

- Either $T_n$ or $T_m$ is bounded. Then player $\textrm{II}$ can win the game by choosing the index having the longest extensions (or with unbounded extensions) in *T* as $u_0$, and then choosing *u* so that $|v| \le |u|$.
- Both $T_n$ and $T_m$ are unbounded. Then, whatever $\textrm{II}$ chooses, she can produce a longer sequence in *T* than $\textrm{I}$, again ensuring $|u| \le |v|$.

In both cases, we obtain a contradiction to the fact that $\sigma$ was a winning strategy.

By determinacy for wellfounded binary choice game trees, $\textrm{II}$ has a winning strategy $\tau$. We then define $f: \mathbb {N}\text{}\rightarrow \mathbb {N}\text{}$ as

$$\begin{aligned} f(n) = u_0(\tau (f[n])), \end{aligned}$$

i.e., the answer $u_0$ of $\tau$ to $\textrm{I}$ playing $t = f[n]$, where *f*[*n*] is the string obtained by concatenating *f*(*k*) for $k < n$.

Recall that $\Sigma ^0_1$-induction is one of the axioms of $\textsf{RCA}_0$. We use it to prove that

$$\begin{aligned} \forall n\, \theta (n), \qquad \text {where } \theta (n) = [f[n] \in T \wedge \exists k\, \big ( f[n]^{\smallfrown }k \in T \big )]. \end{aligned}$$

Note that $\theta (n)$ is indeed a $\Sigma ^0_1$ formula, with *T* and $\tau$ as parameters. First $f[0] = u_0(\tau (\emptyset )) \in T$ since *T* is non-empty and otherwise $\textrm{I}$ could defeat this play by playing *v* in the tree. Moreover, *f*[0] has a successor in the tree. Otherwise, since *T* is infinite (in particular $|T| > 3$), $\textrm{I}$ could have played an extension of $\emptyset$ incompatible with *f*[0] and of length $\ge 2$, defeating a play consistent with $\tau$, which is impossible.

We prove by induction that *f*[*n*] belongs to *T* and has a successor in *T*. We first consider the case $n = 1$ explicitly for the sake of exposition. Since we proved *f*[0] has a successor, $f[1] = f(0)^\smallfrown u_0(\tau (f[0]))$ is in *T*, since, again, otherwise $\textrm{I}$ could defeat $u_0(\tau (f[0]))$ as above by playing *v* in *T*.

Suppose towards a contradiction that

$$\begin{aligned} \lnot \diamond ^1(f[1]) := \forall n \ f[1]^{\smallfrown }n \not \in T \end{aligned}$$

holds; thus $|T_{f[1]}| = 1$. Then because $f[1] = f(0)^\frown u_0(\tau (f[0]))$ and $\tau$ is winning, we have

$$\begin{aligned} \lnot \diamond ^2(f[0]) := \forall n,m \ f[0]^{\smallfrown }\langle n,m \rangle \not \in T, \end{aligned}$$

for otherwise if $v = \langle n,m \rangle$ witnesses $\diamond ^2(f[0])$, then it follows from $\lnot \diamond ^1(f[0])$ that $\textrm{I}$ could have defeated $\tau$ by playing *v* against $u = \langle u_0 \rangle = \langle f(1)\rangle$; thus, $|T_{f[0]}| \le 3$. In a similar way we obtain

$$\begin{aligned} \lnot \diamond ^3(\emptyset ) := \forall n,m,k \ \langle n,m,k \rangle \not \in T. \end{aligned}$$

**Fig. 1:**
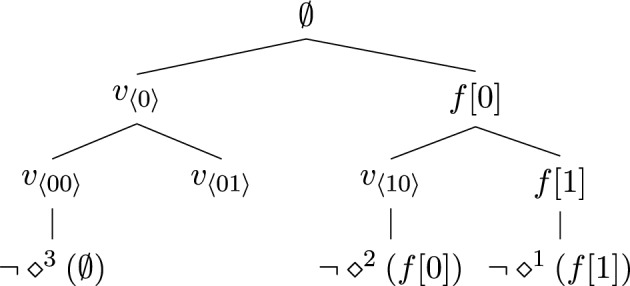
Proof that *f*[1] has a successor in *T*

This is because otherwise if $v = \langle n,m, k\rangle$ witnesses $\diamond ^3(\emptyset )$, then it follows from $\lnot \diamond ^2(f[0] )$ that $\textrm{I}$ could have defeated $\tau$ by playing *v* after $\tau$ plays $u_0 = f[0]$; hence $|T| = |T_\emptyset | \le 7$. But this is a contradiction to the assumption that *T* is infinite, hence the conclusion follows. See Figure 1 for a picture.

The general case follows a similar line of reasoning. Suppose that $f[n] \in T$ and has a successor in *T*. Hence, $f[n+1] \in T$, otherwise $\tau$ would lose after $\textrm{I}$ plays this successor as *v*(0). Now we need to prove that $f[n+1]$ has a successor in *T*. We claim that:

3.2 $$\begin{aligned} |T_{f[n+1-j]}| \le 2^{j+1}-1. \end{aligned}$$

Observe that (3.2) is equivalent to the assertion “all sequences of length $2^{j+1}$ contain an element not in $T_{f[n+1-j]}$” and thus is a $\Pi ^0_1$ assertion about *j* with *T* and $\tau$ as parameters. We prove (3.2) by a subsidiary $\Pi ^0_1$-induction on $j \le n+1$. The base case asserts that $|T_{f[n+1]}| \le 1$ and is precisely the assumption that $f[n+1]$ has no successor in *T*. The inductive step is established by a similar argument as above, through a process extending that of Figure 1, using the fact that $\tau$ is a winning strategy. Thus, by $\Pi ^0_1$-induction, we derive the claim (3.2) which in particular implies that $|T| \le 2^{n+2}-1$, contradicting the assumption that *T* was infinite. Hence, we conclude that $f[n+1]$ indeed has a successor in *T*. By the main $\Sigma ^0_1$-induction, we conclude $\forall n \ f[n] \in T$, showing that *T* has a branch, which is a contradiction. This completes the proof. $\square$

## Data Availability

No datasets were generated or analysed during the current study.
